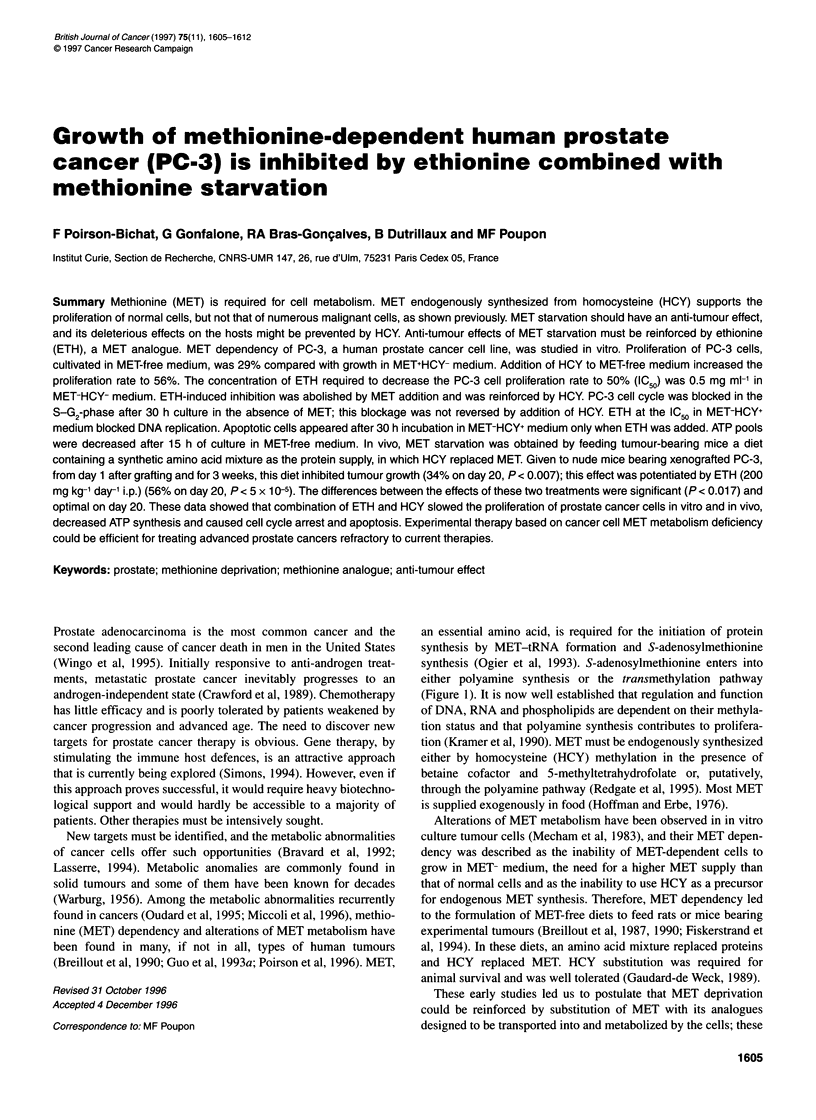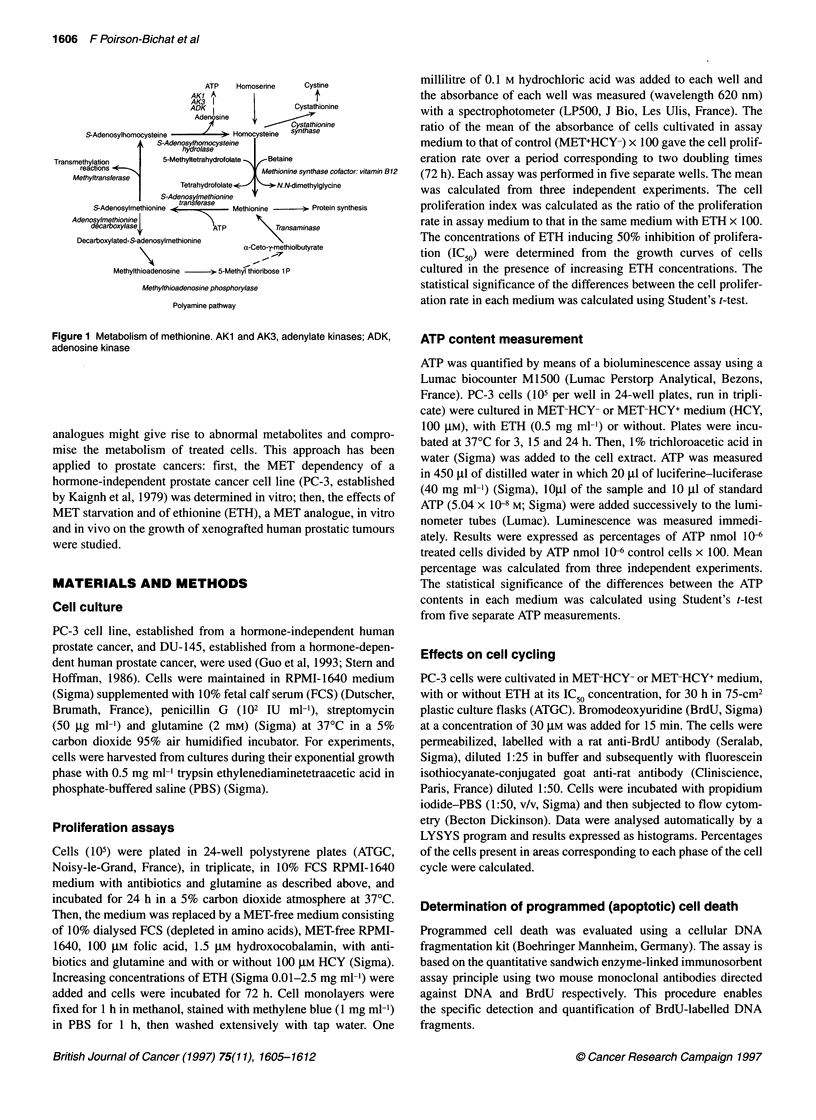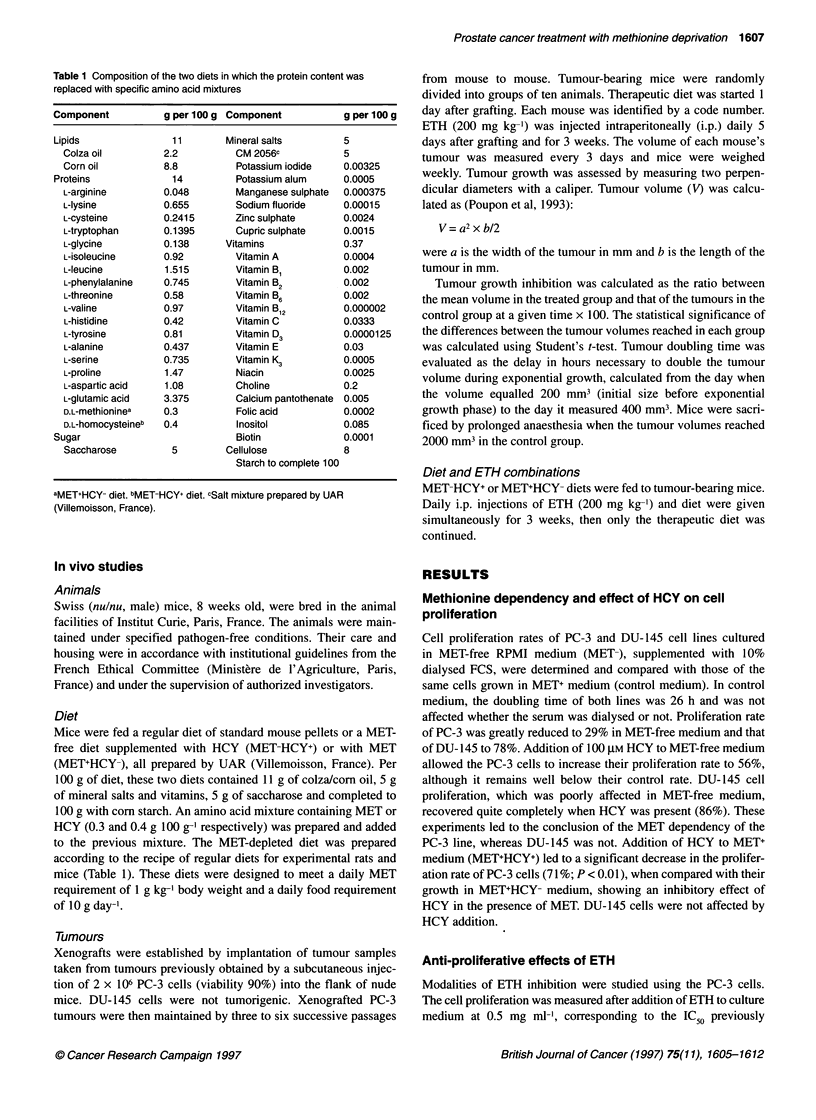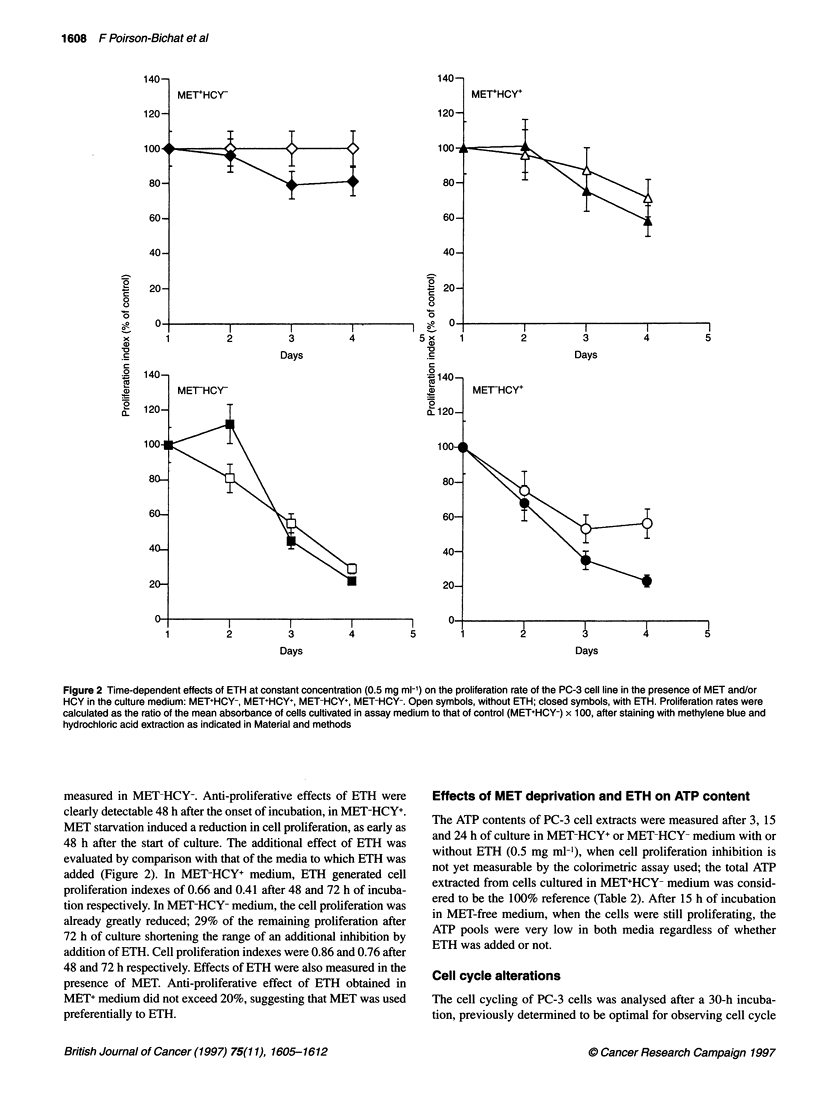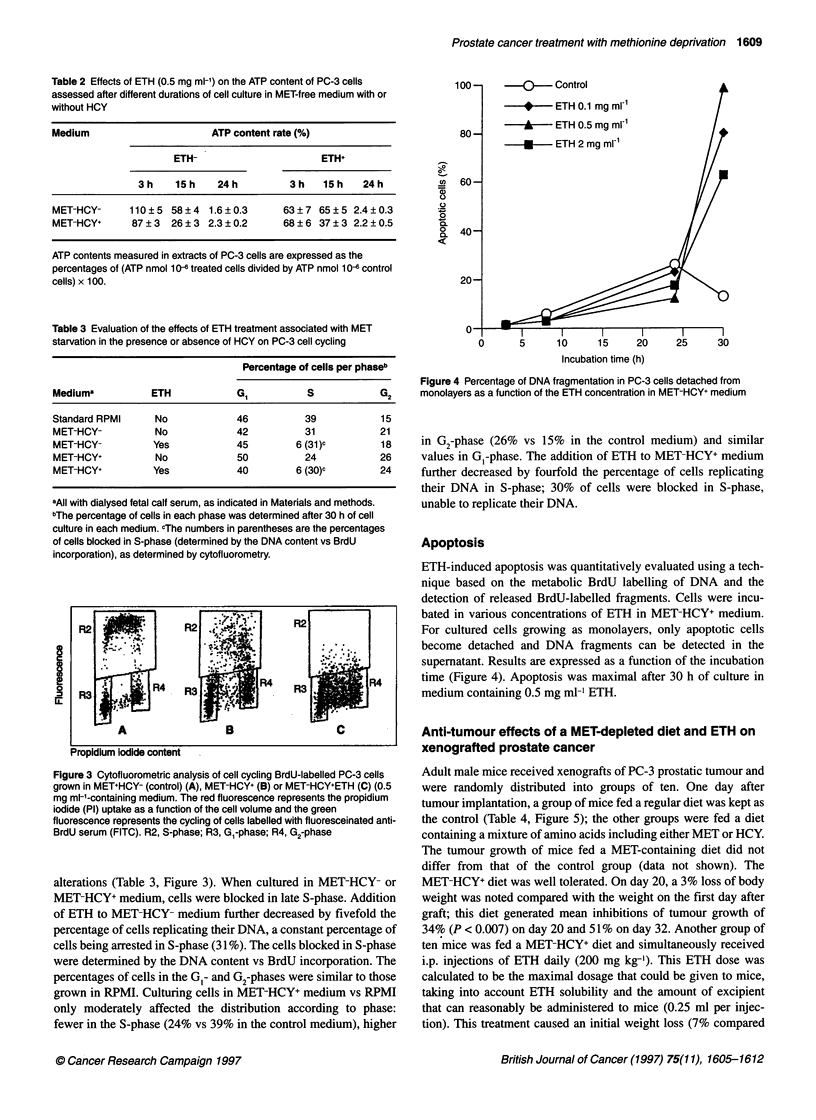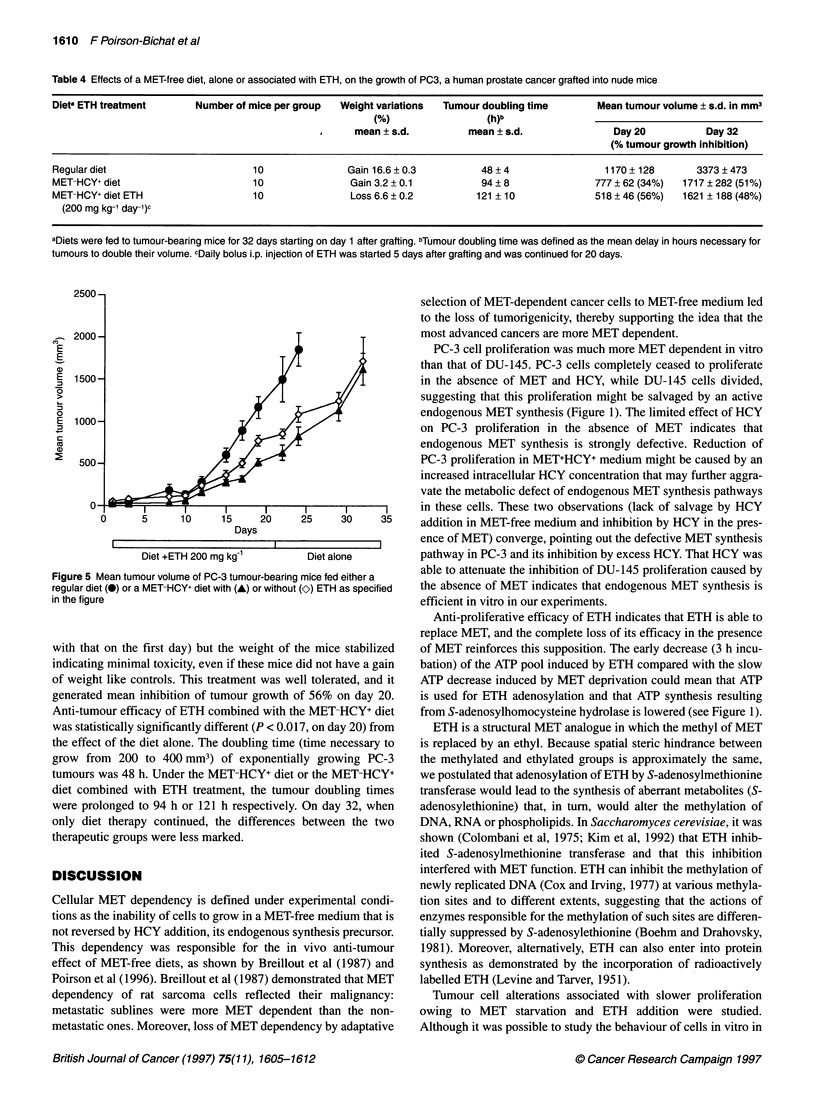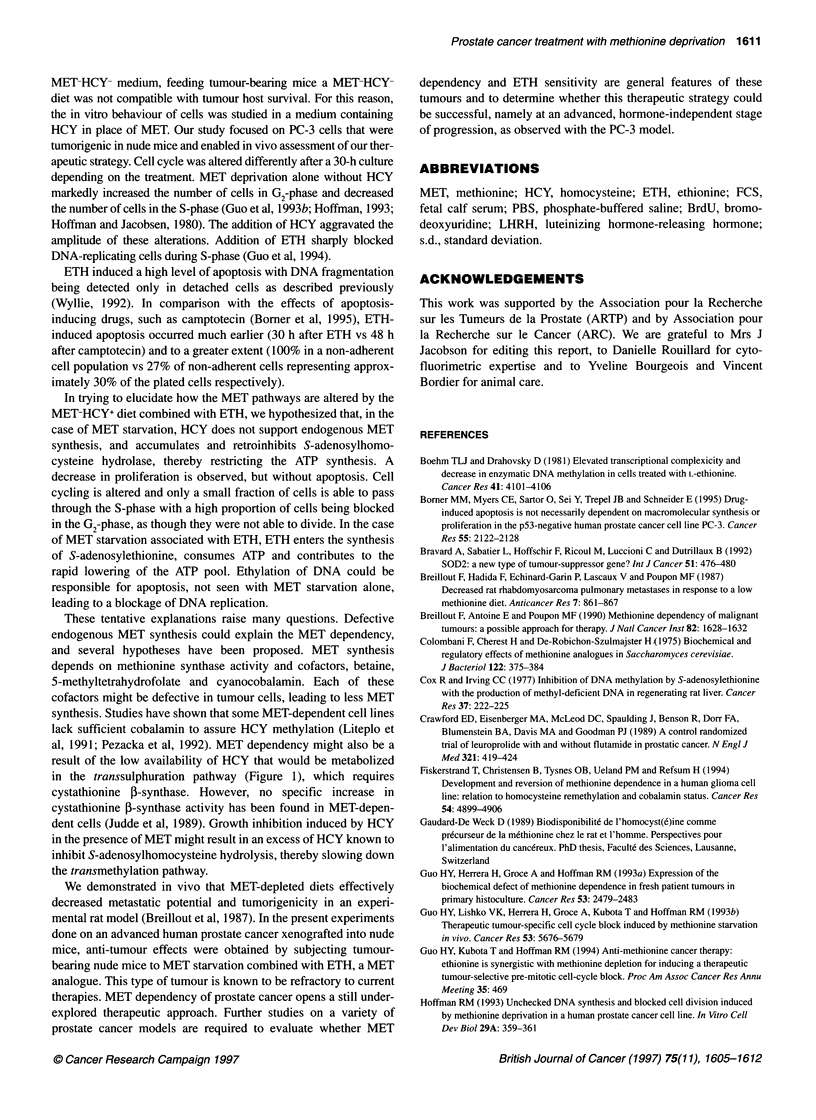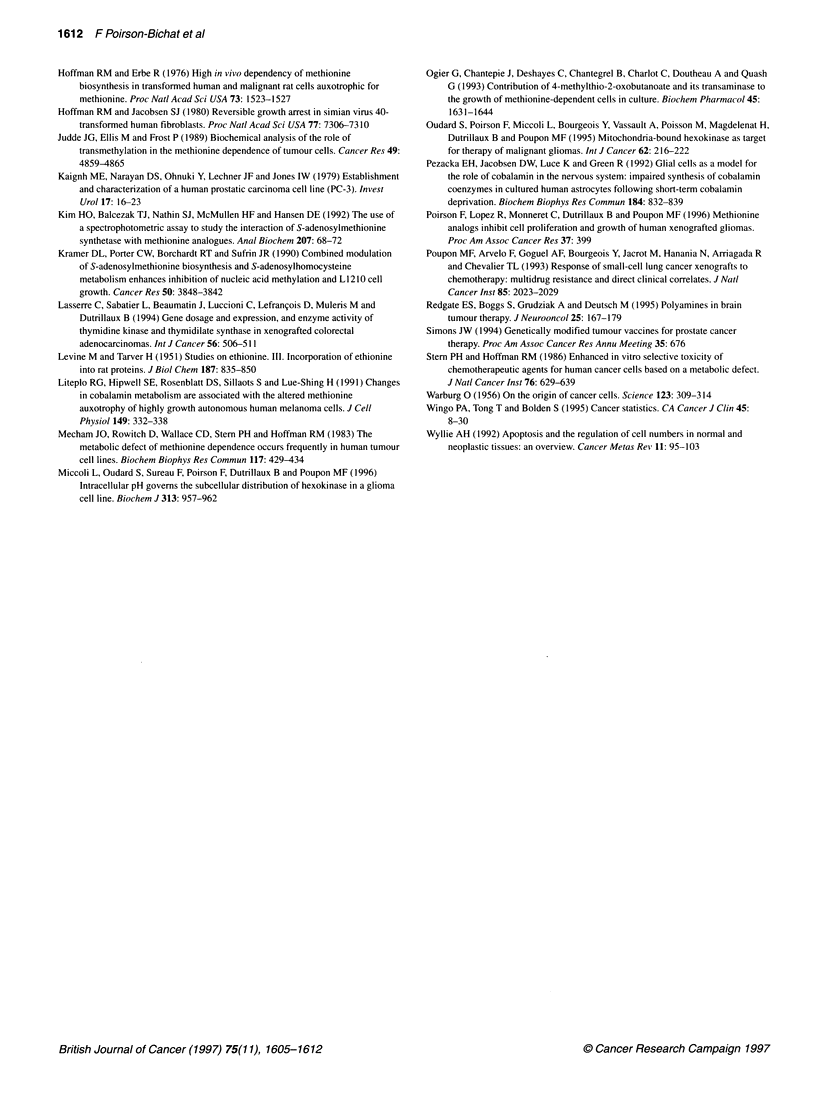# Growth of methionine-dependent human prostate cancer (PC-3) is inhibited by ethionine combined with methionine starvation.

**DOI:** 10.1038/bjc.1997.274

**Published:** 1997

**Authors:** F. Poirson-Bichat, G. Gonfalone, R. A. Bras-GonÃ§alves, B. Dutrillaux, M. F. Poupon

**Affiliations:** Institut Curie, CNRS-UMR 147, Paris, France.

## Abstract

Methionine (MET) is required for cell metabolism. MET endogenously synthesized from homocysteine (HCY) supports the proliferation of normal cells, but not that of numerous malignant cells, as shown previously. MET starvation should have an anti-tumour effect, and its deleterious effects on the hosts might be prevented by HCY. Anti-tumour effects of MET starvation must be reinforced by ethionine (ETH), a MET analogue. MET dependency of PC-3, a human prostate cancer cell line, was studied in vitro. Proliferation of PC-3 cells, cultivated in MET-free medium, was 29% compared with growth in MET+HCY- medium. Addition of HCY to MET-free medium increased the proliferation rate to 56%. The concentration of ETH required to decrease the PC-3 cell proliferation rate to 50% (IC50) was 0.5 mg ml(-1) in MET-HCY- medium. ETH-induced inhibition was abolished by MET addition and was reinforced by HCY. PC-3 cell cycle was blocked in the S-G2-phase after 30 h culture in the absence of MET; this blockage was not reversed by addition of HCY. ETH at the IC50 in MET-HCY+ medium blocked DNA replication. Apoptotic cells appeared after 30 h incubation in MET-HCY+ medium only when ETH was added. ATP pools were decreased after 15 h of culture in MET-free medium. In vivo, MET starvation was obtained by feeding tumour-bearing mice a diet containing a synthetic amino acid mixture as the protein supply, in which HCY replaced MET. Given to nude mice bearing xenografted PC-3, from day 1 after grafting and for 3 weeks, this diet inhibited tumour growth (34% on day 20, P < 0.007); this effect was potentiated by ETH (200 mg kg(-1) day(-1) i.p.) (56% on day 20, P < 5 x 10(-5)). The differences between the effects of these two treatments were significant (P < 0.017) and optimal on day 20. These data showed that combination of ETH and HCY slowed the proliferation of prostate cancer cells in vitro and in vivo, decreased ATP synthesis and caused cell cycle arrest and apoptosis. Experimental therapy based on cancer cell MET metabolism deficiency could be efficient for treating advanced prostate cancers refractory to current therapies.


British Journal of Cancer (1997) 75(11), 1605–1612
© 1997 Cancer Research Campaign

### Growth of methionine-dependent human prostate cancer (PC-3) is inhibited by ethionine combined with methionine starvation

**F Poirson-Bichat, G Gonfalone, RA Bras-Gonçalves, B Dutrillaux and MF Poupon**

Institut Curie, Section de Recherche, CNRS-UMR 147, 26, rue d'Ulm, 75231 Paris Cedex 05, France

**Summary** Methionine (MET) is required for cell metabolism. MET endogenously synthesized from homocysteine (HCY) supports the proliferation of normal cells, but not that of numerous malignant cells, as shown previously. MET starvation should have an anti-tumour effect, and its deleterious effects on the hosts might be prevented by HCY. Anti-tumour effects of MET starvation must be reinforced by ethionine (ETH), a MET analogue. MET dependency of PC-3, a human prostate cancer cell line, was studied in vitro. Proliferation of PC-3 cells, cultivated in MET-free medium, was 29% compared with growth in $MET^{+}HCY^{-}$ medium. Addition of HCY to MET-free medium increased the proliferation rate to 56%. The concentration of ETH required to decrease the PC-3 cell proliferation rate to 50% ($IC_{50}$) was 0.5 mg $ml^{-1}$ in $MET^{-}HCY^{-}$ medium. ETH-induced inhibition was abolished by MET addition and was reinforced by HCY. PC-3 cell cycle was blocked in the S–$G_2$-phase after 30 h culture in the absence of MET; this blockage was not reversed by addition of HCY. ETH at the $IC_{50}$ in $MET^{-}HCY^{+}$ medium blocked DNA replication. Apoptotic cells appeared after 30 h incubation in $MET^{-}HCY^{+}$ medium only when ETH was added. ATP pools were decreased after 15 h of culture in MET-free medium. In vivo, MET starvation was obtained by feeding tumour-bearing mice a diet containing a synthetic amino acid mixture as the protein supply, in which HCY replaced MET. Given to nude mice bearing xenografted PC-3, from day 1 after grafting and for 3 weeks, this diet inhibited tumour growth (34% on day 20, $P < 0.007$); this effect was potentiated by ETH (200 mg $kg^{-1}$ $day^{-1}$ i.p.) (56% on day 20, $P < 5 \times 10^{-5}$). The differences between the effects of these two treatments were significant ($P < 0.017$) and optimal on day 20. These data showed that combination of ETH and HCY slowed the proliferation of prostate cancer cells in vitro and in vivo, decreased ATP synthesis and caused cell cycle arrest and apoptosis. Experimental therapy based on cancer cell MET metabolism deficiency could be efficient for treating advanced prostate cancers refractory to current therapies.

**Keywords:** prostate; methionine deprivation; methionine analogue; anti-tumour effect

Prostate adenocarcinoma is the most common cancer and the second leading cause of cancer death in men in the United States (Wingo et al, 1995). Initially responsive to anti-androgen treatments, metastatic prostate cancer inevitably progresses to an androgen-independent state (Crawford et al, 1989). Chemotherapy has little efficacy and is poorly tolerated by patients weakened by cancer progression and advanced age. The need to discover new targets for prostate cancer therapy is obvious. Gene therapy, by stimulating the immune host defences, is an attractive approach that is currently being explored (Simons, 1994). However, even if this approach proves successful, it would require heavy biotechnological support and would hardly be accessible to a majority of patients. Other therapies must be intensively sought.

New targets must be identified, and the metabolic abnormalities of cancer cells offer such opportunities (Bravard et al, 1992; Lasserre, 1994). Metabolic anomalies are commonly found in solid tumours and some of them have been known for decades (Warburg, 1956). Among the metabolic abnormalities recurrently found in cancers (Oudard et al, 1995; Miccoli et al, 1996), methionine (MET) dependency and alterations of MET metabolism have been found in many, if not in all, types of human tumours (Breillout et al, 1990; Guo et al, 1993*a*; Poirson et al, 1996). MET, an essential amino acid, is required for the initiation of protein synthesis by MET–tRNA formation and *S*-adenosylmethionine synthesis (Ogier et al, 1993). *S*-adenosylmethionine enters into either polyamine synthesis or the *trans*methylation pathway (Figure 1). It is now well established that regulation and function of DNA, RNA and phospholipids are dependent on their methylation status and that polyamine synthesis contributes to proliferation (Kramer et al, 1990). MET must be endogenously synthesized either by homocysteine (HCY) methylation in the presence of betaine cofactor and 5-methyltetrahydrofolate or, putatively, through the polyamine pathway (Redgate et al, 1995). Most MET is supplied exogenously in food (Hoffman and Erbe, 1976).

Alterations of MET metabolism have been observed in in vitro culture tumour cells (Mecham et al, 1983), and their MET dependency was described as the inability of MET-dependent cells to grow in $MET^{-}$ medium, the need for a higher MET supply than that of normal cells and as the inability to use HCY as a precursor for endogenous MET synthesis. Therefore, MET dependency led to the formulation of MET-free diets to feed rats or mice bearing experimental tumours (Breillout et al, 1987, 1990; Fiskerstrand et al, 1994). In these diets, an amino acid mixture replaced proteins and HCY replaced MET. HCY substitution was required for animal survival and was well tolerated (Gaudard-de Weck, 1989).

These early studies led us to postulate that MET deprivation could be reinforced by substitution of MET with its analogues designed to be transported into and metabolized by the cells; these

*Revised 31 October 1996*
*Accepted 4 December 1996*

*Correspondence to:* MF Poupon

**Figure 1** Metabolism of methionine. AK1 and AK3, adenylate kinases; ADK, adenosine kinase analogues might give rise to abnormal metabolites and compromise the metabolism of treated cells. This approach has been applied to prostate cancers: first, the MET dependency of a hormone-independent prostate cancer cell line (PC-3, established by Kaignh et al, 1979) was determined in vitro; then, the effects of MET starvation and of ethionine (ETH), a MET analogue, in vitro and in vivo on the growth of xenografted human prostatic tumours were studied.

### MATERIALS AND METHODS

### Cell culture

PC-3 cell line, established from a hormone-independent human prostate cancer, and DU-145, established from a hormone-dependent human prostate cancer, were used (Guo et al, 1993; Stern and Hoffman, 1986). Cells were maintained in RPMI-1640 medium (Sigma) supplemented with 10% fetal calf serum (FCS) (Dutscher, Brumath, France), penicillin G ($10^2$ IU ml$^{-1}$), streptomycin (50 μg ml$^{-1}$) and glutamine (2 mM) (Sigma) at 37°C in a 5% carbon dioxide 95% air humidified incubator. For experiments, cells were harvested from cultures during their exponential growth phase with 0.5 mg ml$^{-1}$ trypsin ethylenediaminetetraacetic acid in phosphate-buffered saline (PBS) (Sigma).

### Proliferation assays

Cells ($10^5$) were plated in 24-well polystyrene plates (ATGC, Noisy-le-Grand, France), in triplicate, in 10% FCS RPMI-1640 medium with antibiotics and glutamine as described above, and incubated for 24 h in a 5% carbon dioxide atmosphere at 37°C. Then, the medium was replaced by a MET-free medium consisting of 10% dialysed FCS (depleted in amino acids), MET-free RPMI-1640, 100 μM folic acid, 1.5 μM hydroxocobalamin, with antibiotics and glutamine and with or without 100 μM HCY (Sigma). Increasing concentrations of ETH (Sigma 0.01–2.5 mg ml$^{-1}$) were added and cells were incubated for 72 h. Cell monolayers were fixed for 1 h in methanol, stained with methylene blue (1 mg ml$^{-1}$) in PBS for 1 h, then washed extensively with tap water. One millilitre of 0.1 M hydrochloric acid was added to each well and the absorbance of each well was measured (wavelength 620 nm) with a spectrophotometer (LP500, J Bio, Les Ulis, France). The ratio of the mean of the absorbance of cells cultivated in assay medium to that of control ($MET^+HCY^-$) × 100 gave the cell proliferation rate over a period corresponding to two doubling times (72 h). Each assay was performed in five separate wells. The mean was calculated from three independent experiments. The cell proliferation index was calculated as the ratio of the proliferation rate in assay medium to that in the same medium with ETH × 100. The concentrations of ETH inducing 50% inhibition of proliferation ($IC_{50}$) were determined from the growth curves of cells cultured in the presence of increasing ETH concentrations. The statistical significance of the differences between the cell proliferation rate in each medium was calculated using Student's *t*-test.

### ATP content measurement

ATP was quantified by means of a bioluminescence assay using a Lumac biocounter M1500 (Lumac Perstorp Analytical, Bezons, France). PC-3 cells ($10^5$ per well in 24-well plates, run in triplicate) were cultured in $MET^-HCY^-$ or $MET^-HCY^+$ medium (HCY, 100 μM), with ETH (0.5 mg ml$^{-1}$) or without. Plates were incubated at 37°C for 3, 15 and 24 h. Then, 1% trichloroacetic acid in water (Sigma) was added to the cell extract. ATP was measured in 450 μl of distilled water in which 20 μl of luciferine–luciferase (40 mg ml$^{-1}$) (Sigma), 10μl of the sample and 10 μl of standard ATP ($5.04 \times 10^{-8}$ M; Sigma) were added successively to the luminometer tubes (Lumac). Luminescence was measured immediately. Results were expressed as percentages of ATP nmol $10^{-6}$ treated cells divided by ATP nmol $10^{-6}$ control cells × 100. Mean percentage was calculated from three independent experiments. The statistical significance of the differences between the ATP contents in each medium was calculated using Student's *t*-test from five separate ATP measurements.

### Effects on cell cycling

PC-3 cells were cultivated in $MET^-HCY^-$ or $MET^-HCY^+$ medium, with or without ETH at its $IC_{50}$ concentration, for 30 h in 75-cm$^2$ plastic culture flasks (ATGC). Bromodeoxyuridine (BrdU, Sigma) at a concentration of 30 μM was added for 15 min. The cells were permeabilized, labelled with a rat anti-BrdU antibody (Seralab, Sigma), diluted 1:25 in buffer and subsequently with fluorescein isothiocyanate-conjugated goat anti-rat antibody (Cliniscience, Paris, France) diluted 1:50. Cells were incubated with propidium iodide–PBS (1:50, v/v, Sigma) and then subjected to flow cytometry (Becton Dickinson). Data were analysed automatically by a LYSYS program and results expressed as histograms. Percentages of the cells present in areas corresponding to each phase of the cell cycle were calculated.

### Determination of programmed (apoptotic) cell death

Programmed cell death was evaluated using a cellular DNA fragmentation kit (Boehringer Mannheim, Germany). The assay is based on the quantitative sandwich enzyme-linked immunosorbent assay principle using two mouse monoclonal antibodies directed against DNA and BrdU respectively. This procedure enables the specific detection and quantification of BrdU-labelled DNA fragments.

© Cancer Research Campaign 1997

**Table 1** Composition of the two diets in which the protein content was replaced with specific amino acid mixtures

| Component | g per 100 g | Component | g per 100 g |
|---|---|---|---|
| Lipids | 11 | Mineral salts | 5 |
| Colza oil | 2.2 | CM 2056[c] | 5 |
| Corn oil | 8.8 | Potassium iodide | 0.00325 |
| Proteins | 14 | Potassium alum | 0.0005 |
| L-arginine | 0.048 | Manganese sulphate | 0.000375 |
| L-lysine | 0.655 | Sodium fluoride | 0.00015 |
| L-cysteine | 0.2415 | Zinc sulphate | 0.0024 |
| L-tryptophan | 0.1395 | Cupric sulphate | 0.0015 |
| L-glycine | 0.138 | Vitamins | 0.37 |
| L-isoleucine | 0.92 | Vitamin A | 0.0004 |
| L-leucine | 1.515 | Vitamin $B_1$ | 0.002 |
| L-phenylalanine | 0.745 | Vitamin $B_2$ | 0.002 |
| L-threonine | 0.58 | Vitamin $B_6$ | 0.002 |
| L-valine | 0.97 | Vitamin $B_{12}$ | 0.000002 |
| L-histidine | 0.42 | Vitamin C | 0.0333 |
| L-tyrosine | 0.81 | Vitamin $D_3$ | 0.0000125 |
| L-alanine | 0.437 | Vitamin E | 0.03 |
| L-serine | 0.735 | Vitamin $K_3$ | 0.0005 |
| L-proline | 1.47 | Niacin | 0.0025 |
| L-aspartic acid | 1.08 | Choline | 0.2 |
| L-glutamic acid | 3.375 | Calcium pantothenate | 0.005 |
| D.L-methionine[a] | 0.3 | Folic acid | 0.0002 |
| D.L-homocysteine[b] | 0.4 | Inositol | 0.085 |
| Sugar | | Biotin | 0.0001 |
| Saccharose | 5 | Cellulose | 8 |
| | | Starch to complete 100 | |

[a]$MET^+HCY^-$ diet. [b]$MET^-HCY^+$ diet. [c]Salt mixture prepared by UAR (Villemoisson, France).

### In vivo studies

#### *Animals*

Swiss (*nu/nu*, male) mice, 8 weeks old, were bred in the animal facilities of Institut Curie, Paris, France. The animals were maintained under specified pathogen-free conditions. Their care and housing were in accordance with institutional guidelines from the French Ethical Committee (Ministère de l'Agriculture, Paris, France) and under the supervision of authorized investigators.

#### *Diet*

Mice were fed a regular diet of standard mouse pellets or a MET-free diet supplemented with HCY ($MET^-HCY^+$) or with MET ($MET^+HCY^-$), all prepared by UAR (Villemoisson, France). Per 100 g of diet, these two diets contained 11 g of colza/corn oil, 5 g of mineral salts and vitamins, 5 g of saccharose and completed to 100 g with corn starch. An amino acid mixture containing MET or HCY (0.3 and 0.4 g 100 $g^{-1}$ respectively) was prepared and added to the previous mixture. The MET-depleted diet was prepared according to the recipe of regular diets for experimental rats and mice (Table 1). These diets were designed to meet a daily MET requirement of 1 g $kg^{-1}$ body weight and a daily food requirement of 10 g $day^{-1}$.

#### *Tumours*

Xenografts were established by implantation of tumour samples taken from tumours previously obtained by a subcutaneous injection of $2 \times 10^6$ PC-3 cells (viability 90%) into the flank of nude mice. DU-145 cells were not tumorigenic. Xenografted PC-3 tumours were then maintained by three to six successive passages from mouse to mouse. Tumour-bearing mice were randomly divided into groups of ten animals. Therapeutic diet was started 1 day after grafting. Each mouse was identified by a code number. ETH (200 mg $kg^{-1}$) was injected intraperitoneally (i.p.) daily 5 days after grafting and for 3 weeks. The volume of each mouse's tumour was measured every 3 days and mice were weighed weekly. Tumour growth was assessed by measuring two perpendicular diameters with a caliper. Tumour volume ($V$) was calculated as (Poupon et al, 1993):

$$V = a^2 \times b/2$$

were $a$ is the width of the tumour in mm and $b$ is the length of the tumour in mm.

Tumour growth inhibition was calculated as the ratio between the mean volume in the treated group and that of the tumours in the control group at a given time × 100. The statistical significance of the differences between the tumour volumes reached in each group was calculated using Student's $t$-test. Tumour doubling time was evaluated as the delay in hours necessary to double the tumour volume during exponential growth, calculated from the day when the volume equalled 200 $mm^3$ (initial size before exponential growth phase) to the day it measured 400 $mm^3$. Mice were sacrificed by prolonged anaesthesia when the tumour volumes reached 2000 $mm^3$ in the control group.

#### *Diet and ETH combinations*

$MET^-HCY^+$ or $MET^+HCY^-$ diets were fed to tumour-bearing mice. Daily i.p. injections of ETH (200 mg $kg^{-1}$) and diet were given simultaneously for 3 weeks, then only the therapeutic diet was continued.

### RESULTS

### Methionine dependency and effect of HCY on cell proliferation

Cell proliferation rates of PC-3 and DU-145 cell lines cultured in MET-free RPMI medium ($MET^-$), supplemented with 10% dialysed FCS, were determined and compared with those of the same cells grown in $MET^+$ medium (control medium). In control medium, the doubling time of both lines was 26 h and was not affected whether the serum was dialysed or not. Proliferation rate of PC-3 was greatly reduced to 29% in MET-free medium and that of DU-145 to 78%. Addition of 100 μM HCY to MET-free medium allowed the PC-3 cells to increase their proliferation rate to 56%, although it remains well below their control rate. DU-145 cell proliferation, which was poorly affected in MET-free medium, recovered quite completely when HCY was present (86%). These experiments led to the conclusion of the MET dependency of the PC-3 line, whereas DU-145 was not. Addition of HCY to $MET^+$ medium ($MET^+HCY^+$) led to a significant decrease in the proliferation rate of PC-3 cells (71%; $P < 0.01$), when compared with their growth in $MET^+HCY^-$ medium, showing an inhibitory effect of HCY in the presence of MET. DU-145 cells were not affected by HCY addition.

### Anti-proliferative effects of ETH

Modalities of ETH inhibition were studied using the PC-3 cells. The cell proliferation was measured after addition of ETH to culture medium at 0.5 mg $ml^{-1}$, corresponding to the $IC_{50}$ previously

**Figure 2** Time-dependent effects of ETH at constant concentration (0.5 mg $ml^{-1}$) on the proliferation rate of the PC-3 cell line in the presence of MET and/or HCY in the culture medium: $MET^{+}HCY^{-}$, $MET^{+}HCY^{+}$, $MET^{-}HCY^{+}$, $MET^{-}HCY^{-}$. Open symbols, without ETH; closed symbols, with ETH. Proliferation rates were calculated as the ratio of the mean absorbance of cells cultivated in assay medium to that of control ($MET^{+}HCY^{-}$) x 100, after staining with methylene blue and hydrochloric acid extraction as indicated in Material and methods measured in $MET^{-}HCY^{-}$. Anti-proliferative effects of ETH were clearly detectable 48 h after the onset of incubation, in $MET^{-}HCY^{+}$. MET starvation induced a reduction in cell proliferation, as early as 48 h after the start of culture. The additional effect of ETH was evaluated by comparison with that of the media to which ETH was added (Figure 2). In $MET^{-}HCY^{+}$ medium, ETH generated cell proliferation indexes of 0.66 and 0.41 after 48 and 72 h of incubation respectively. In $MET^{-}HCY^{-}$ medium, the cell proliferation was already greatly reduced; 29% of the remaining proliferation after 72 h of culture shortening the range of an additional inhibition by addition of ETH. Cell proliferation indexes were 0.86 and 0.76 after 48 and 72 h respectively. Effects of ETH were also measured in the presence of MET. Anti-proliferative effect of ETH obtained in $MET^{+}$ medium did not exceed 20%, suggesting that MET was used preferentially to ETH.

### Effects of MET deprivation and ETH on ATP content

The ATP contents of PC-3 cell extracts were measured after 3, 15 and 24 h of culture in $MET^{-}HCY^{+}$ or $MET^{-}HCY^{-}$ medium with or without ETH (0.5 mg $ml^{-1}$), when cell proliferation inhibition is not yet measurable by the colorimetric assay used; the total ATP extracted from cells cultured in $MET^{+}HCY^{-}$ medium was considered to be the 100% reference (Table 2). After 15 h of incubation in MET-free medium, when the cells were still proliferating, the ATP pools were very low in both media regardless of whether ETH was added or not.

### Cell cycle alterations

The cell cycling of PC-3 cells was analysed after a 30-h incubation, previously determined to be optimal for observing cell cycle

© Cancer Research Campaign 1997

**Table 2** Effects of ETH (0.5 mg ml$^{-1}$) on the ATP content of PC-3 cells assessed after different durations of cell culture in MET-free medium with or without HCY

| Medium | ATP content rate (%) | | | | | |
|---|---|---|---|---|---|---|
| | ETH$^-$ | | | ETH$^+$ | | |
| | 3 h | 15 h | 24 h | 3 h | 15 h | 24 h |
| MET$^-$HCY$^-$ | 110 ± 5 | 58 ± 4 | 1.6 ± 0.3 | 63 ± 7 | 65 ± 5 | 2.4 ± 0.3 |
| MET$^-$HCY$^+$ | 87 ± 3 | 26 ± 3 | 2.3 ± 0.2 | 68 ± 6 | 37 ± 3 | 2.2 ± 0.5 |

ATP contents measured in extracts of PC-3 cells are expressed as the percentages of (ATP nmol $10^{-6}$ treated cells divided by ATP nmol $10^{-6}$ control cells) × 100.

**Table 3** Evaluation of the effects of ETH treatment associated with MET starvation in the presence or absence of HCY on PC-3 cell cycling

| Medium[a] | ETH | Percentage of cells per phase[b] | | |
|---|---|---|---|---|
| | | $G_1$ | S | $G_2$ |
| Standard RPMI | No | 46 | 39 | 15 |
| MET$^-$HCY$^-$ | No | 42 | 31 | 21 |
| MET$^-$HCY$^-$ | Yes | 45 | 6 (31)[c] | 18 |
| MET$^-$HCY$^+$ | No | 50 | 24 | 26 |
| MET$^-$HCY$^+$ | Yes | 40 | 6 (30)[c] | 24 |

[a]All with dialysed fetal calf serum, as indicated in Materials and methods. [b]The percentage of cells in each phase was determined after 30 h of cell culture in each medium. [c]The numbers in parentheses are the percentages of cells blocked in S-phase (determined by the DNA content vs BrdU incorporation), as determined by cytofluorometry.

**Figure 3** Cytofluorometric analysis of cell cycling BrdU-labelled PC-3 cells grown in MET$^+$HCY$^-$ (control) (**A**), MET$^-$HCY$^+$ (**B**) or MET$^-$HCY$^+$ETH (**C**) (0.5 mg ml$^{-1}$-containing medium. The red fluorescence represents the propidium iodide (PI) uptake as a function of the cell volume and the green fluorescence represents the cycling of cells labelled with fluoresceinated anti-BrdU serum (FITC). R2, S-phase; R3, $G_1$-phase; R4, $G_2$-phase alterations (Table 3, Figure 3). When cultured in MET$^-$HCY$^-$ or MET$^-$HCY$^+$ medium, cells were blocked in late S-phase. Addition of ETH to MET$^-$HCY$^-$ medium further decreased by fivefold the percentage of cells replicating their DNA, a constant percentage of cells being arrested in S-phase (31%). The cells blocked in S-phase were determined by the DNA content vs BrdU incorporation. The percentages of cells in the $G_1$- and $G_2$-phases were similar to those grown in RPMI. Culturing cells in MET$^-$HCY$^+$ medium vs RPMI only moderately affected the distribution according to phase: fewer in the S-phase (24% vs 39% in the control medium), higher

**Figure 4** Percentage of DNA fragmentation in PC-3 cells detached from monolayers as a function of the ETH concentration in MET$^-$HCY$^+$ medium in $G_2$-phase (26% vs 15% in the control medium) and similar values in $G_1$-phase. The addition of ETH to MET$^-$HCY$^+$ medium further decreased by fourfold the percentage of cells replicating their DNA in S-phase; 30% of cells were blocked in S-phase, unable to replicate their DNA.

### Apoptosis

ETH-induced apoptosis was quantitatively evaluated using a technique based on the metabolic BrdU labelling of DNA and the detection of released BrdU-labelled fragments. Cells were incubated in various concentrations of ETH in MET$^-$HCY$^+$ medium. For cultured cells growing as monolayers, only apoptotic cells become detached and DNA fragments can be detected in the supernatant. Results are expressed as a function of the incubation time (Figure 4). Apoptosis was maximal after 30 h of culture in medium containing 0.5 mg ml$^{-1}$ ETH.

### Anti-tumour effects of a MET-depleted diet and ETH on xenografted prostate cancer

Adult male mice received xenografts of PC-3 prostatic tumour and were randomly distributed into groups of ten. One day after tumour implantation, a group of mice fed a regular diet was kept as the control (Table 4, Figure 5); the other groups were fed a diet containing a mixture of amino acids including either MET or HCY. The tumour growth of mice fed a MET-containing diet did not differ from that of the control group (data not shown). The MET$^-$HCY$^+$ diet was well tolerated. On day 20, a 3% loss of body weight was noted compared with the weight on the first day after graft; this diet generated mean inhibitions of tumour growth of 34% ($P < 0.007$) on day 20 and 51% on day 32. Another group of ten mice was fed a MET$^-$HCY$^+$ diet and simultaneously received i.p. injections of ETH daily (200 mg kg$^{-1}$). This ETH dose was calculated to be the maximal dosage that could be given to mice, taking into account ETH solubility and the amount of excipient that can reasonably be administered to mice (0.25 ml per injection). This treatment caused an initial weight loss (7% compared

© Cancer Research Campaign 1997

Table 4 Effects of a MET-free diet, alone or associated with ETH, on the growth of PC3, a human prostate cancer grafted into nude mice

| Diet[a] ETH treatment | Number of mice per group | Weight variations (%) mean ± s.d. | Tumour doubling time (h)[b] mean ± s.d. | Mean tumour volume ± s.d. in mm³ Day 20 (% tumour growth inhibition) | Day 32 |
|---|---|---|---|---|---|
| Regular diet | 10 | Gain 16.6 ± 0.3 | 48 ± 4 | 1170 ± 128 | 3373 ± 473 |
| MET⁻HCY⁺ diet | 10 | Gain 3.2 ± 0.1 | 94 ± 8 | 777 ± 62 (34%) | 1717 ± 282 (51%) |
| MET⁻HCY⁺ diet ETH (200 mg kg⁻¹ day⁻¹)[c] | 10 | Loss 6.6 ± 0.2 | 121 ± 10 | 518 ± 46 (56%) | 1621 ± 188 (48%) |

[a]Diets were fed to tumour-bearing mice for 32 days starting on day 1 after grafting. [b]Tumour doubling time was defined as the mean delay in hours necessary for tumours to double their volume. [c]Daily bolus i.p. injection of ETH was started 5 days after grafting and was continued for 20 days.

Figure 5 Mean tumour volume of PC-3 tumour-bearing mice fed either a regular diet (●) or a MET⁻HCY⁺ diet with (▲) or without (◇) ETH as specified in the figure with that on the first day) but the weight of the mice stabilized indicating minimal toxicity, even if these mice did not have a gain of weight like controls. This treatment was well tolerated, and it generated mean inhibition of tumour growth of 56% on day 20. Anti-tumour efficacy of ETH combined with the MET⁻HCY⁺ diet was statistically significantly different ($P < 0.017$, on day 20) from the effect of the diet alone. The doubling time (time necessary to grow from 200 to 400 mm³) of exponentially growing PC-3 tumours was 48 h. Under the MET⁻HCY⁺ diet or the MET⁻HCY⁺ diet combined with ETH treatment, the tumour doubling times were prolonged to 94 h or 121 h respectively. On day 32, when only diet therapy continued, the differences between the two therapeutic groups were less marked.

### DISCUSSION

Cellular MET dependency is defined under experimental conditions as the inability of cells to grow in a MET-free medium that is not reversed by HCY addition, its endogenous synthesis precursor. This dependency was responsible for the in vivo anti-tumour effect of MET-free diets, as shown by Breillout et al (1987) and Poirson et al (1996). Breillout et al (1987) demonstrated that MET dependency of rat sarcoma cells reflected their malignancy: metastatic sublines were more MET dependent than the non-metastatic ones. Moreover, loss of MET dependency by adaptative selection of MET-dependent cancer cells to MET-free medium led to the loss of tumorigenicity, thereby supporting the idea that the most advanced cancers are more MET dependent.

PC-3 cell proliferation was much more MET dependent in vitro than that of DU-145. PC-3 cells completely ceased to proliferate in the absence of MET and HCY, while DU-145 cells divided, suggesting that this proliferation might be salvaged by an active endogenous MET synthesis (Figure 1). The limited effect of HCY on PC-3 proliferation in the absence of MET indicates that endogenous MET synthesis is strongly defective. Reduction of PC-3 proliferation in MET⁺HCY⁺ medium might be caused by an increased intracellular HCY concentration that may further aggravate the metabolic defect of endogenous MET synthesis pathways in these cells. These two observations (lack of salvage by HCY addition in MET-free medium and inhibition by HCY in the presence of MET) converge, pointing out the defective MET synthesis pathway in PC-3 and its inhibition by excess HCY. That HCY was able to attenuate the inhibition of DU-145 proliferation caused by the absence of MET indicates that endogenous MET synthesis is efficient in vitro in our experiments.

Anti-proliferative efficacy of ETH indicates that ETH is able to replace MET, and the complete loss of its efficacy in the presence of MET reinforces this supposition. The early decrease (3 h incubation) of the ATP pool induced by ETH compared with the slow ATP decrease induced by MET deprivation could mean that ATP is used for ETH adenosylation and that ATP synthesis resulting from *S*-adenosylhomocysteine hydrolase is lowered (see Figure 1).

ETH is a structural MET analogue in which the methyl of MET is replaced by an ethyl. Because spatial steric hindrance between the methylated and ethylated groups is approximately the same, we postulated that adenosylation of ETH by *S*-adenosylmethionine transferase would lead to the synthesis of aberrant metabolites (*S*-adenosylethionine) that, in turn, would alter the methylation of DNA, RNA or phospholipids. In *Saccharomyces cerevisiae*, it was shown (Colombani et al, 1975; Kim et al, 1992) that ETH inhibited *S*-adenosylmethionine transferase and that this inhibition interfered with MET function. ETH can inhibit the methylation of newly replicated DNA (Cox and Irving, 1977) at various methylation sites and to different extents, suggesting that the actions of enzymes responsible for the methylation of such sites are differentially suppressed by *S*-adenosylethionine (Boehm and Drahovsky, 1981). Moreover, alternatively, ETH can also enter into protein synthesis as demonstrated by the incorporation of radioactively labelled ETH (Levine and Tarver, 1951).

Tumour cell alterations associated with slower proliferation owing to MET starvation and ETH addition were studied. Although it was possible to study the behaviour of cells in vitro in

 © Cancer Research Campaign 1997

$MET^-HCY^-$ medium, feeding tumour-bearing mice a $MET^-HCY^-$ diet was not compatible with tumour host survival. For this reason, the in vitro behaviour of cells was studied in a medium containing HCY in place of MET. Our study focused on PC-3 cells that were tumorigenic in nude mice and enabled in vivo assessment of our therapeutic strategy. Cell cycle was altered differently after a 30-h culture depending on the treatment. MET deprivation alone without HCY markedly increased the number of cells in $G_2$-phase and decreased the number of cells in the S-phase (Guo et al, 1993*b*; Hoffman, 1993; Hoffman and Jacobsen, 1980). The addition of HCY aggravated the amplitude of these alterations. Addition of ETH sharply blocked DNA-replicating cells during S-phase (Guo et al, 1994).

ETH induced a high level of apoptosis with DNA fragmentation being detected only in detached cells as described previously (Wyllie, 1992). In comparison with the effects of apoptosis-inducing drugs, such as camptotecin (Borner et al, 1995), ETH-induced apoptosis occurred much earlier (30 h after ETH vs 48 h after camptotecin) and to a greater extent (100% in a non-adherent cell population vs 27% of non-adherent cells representing approximately 30% of the plated cells respectively).

In trying to elucidate how the MET pathways are altered by the $MET^-HCY^+$ diet combined with ETH, we hypothesized that, in the case of MET starvation, HCY does not support endogenous MET synthesis, and accumulates and retroinhibits *S*-adenosylhomocysteine hydrolase, thereby restricting the ATP synthesis. A decrease in proliferation is observed, but without apoptosis. Cell cycling is altered and only a small fraction of cells is able to pass through the S-phase with a high proportion of cells being blocked in the $G_2$-phase, as though they were not able to divide. In the case of MET starvation associated with ETH, ETH enters the synthesis of *S*-adenosylethionine, consumes ATP and contributes to the rapid lowering of the ATP pool. Ethylation of DNA could be responsible for apoptosis, not seen with MET starvation alone, leading to a blockage of DNA replication.

These tentative explanations raise many questions. Defective endogenous MET synthesis could explain the MET dependency, and several hypotheses have been proposed. MET synthesis depends on methionine synthase activity and cofactors, betaine, 5-methyltetrahydrofolate and cyanocobalamin. Each of these cofactors might be defective in tumour cells, leading to less MET synthesis. Studies have shown that some MET-dependent cell lines lack sufficient cobalamin to assure HCY methylation (Liteplo et al, 1991; Pezacka et al, 1992). MET dependency might also be a result of the low availability of HCY that would be metabolized in the *trans*sulphuration pathway (Figure 1), which requires cystathionine β-synthase. However, no specific increase in cystathionine β-synthase activity has been found in MET-dependent cells (Judde et al, 1989). Growth inhibition induced by HCY in the presence of MET might result in an excess of HCY known to inhibit *S*-adenosylhomocysteine hydrolysis, thereby slowing down the *trans*methylation pathway.

We demonstrated in vivo that MET-depleted diets effectively decreased metastatic potential and tumorigenicity in an experimental rat model (Breillout et al, 1987). In the present experiments done on an advanced human prostate cancer xenografted into nude mice, anti-tumour effects were obtained by subjecting tumour-bearing nude mice to MET starvation combined with ETH, a MET analogue. This type of tumour is known to be refractory to current therapies. MET dependency of prostate cancer opens a still under-explored therapeutic approach. Further studies on a variety of prostate cancer models are required to evaluate whether MET dependency and ETH sensitivity are general features of these tumours and to determine whether this therapeutic strategy could be successful, namely at an advanced, hormone-independent stage of progression, as observed with the PC-3 model.

### ABBREVIATIONS

MET, methionine; HCY, homocysteine; ETH, ethionine; FCS, fetal calf serum; PBS, phosphate-buffered saline; BrdU, bromodeoxyuridine; LHRH, luteinizing hormone-releasing hormone; s.d., standard deviation.

### ACKNOWLEDGEMENTS

This work was supported by the Association pour la Recherche sur les Tumeurs de la Prostate (ARTP) and by Association pour la Recherche sur le Cancer (ARC). We are grateful to Mrs J Jacobson for editing this report, to Danielle Rouillard for cytofluorimetric expertise and to Yveline Bourgeois and Vincent Bordier for animal care.

### REFERENCES

Boehm TLJ and Drahovsky D (1981) Elevated transcriptional complexicity and decrease in enzymatic DNA methylation in cells treated with L-ethionine. *Cancer Res* **41**: 4101–4106

Borner MM, Myers CE, Sartor O, Sei Y, Trepel JB and Schneider E (1995) Drug-induced apoptosis is not necessarily dependent on macromolecular synthesis or proliferation in the p53-negative human prostate cancer cell line PC-3. *Cancer Res* **55**: 2122–2128

Bravard A, Sabatier L, Hoffschir F, Ricoul M, Luccioni C and Dutrillaux B (1992) SOD2: a new type of tumour-suppressor gene? *Int J Cancer* **51**: 476–480

Breillout F, Hadida F, Echinard-Garin P, Lascaux V and Poupon MF (1987) Decreased rat rhabdomyosarcoma pulmonary metastases in response to a low methionine diet. *Anticancer Res* **7**: 861–867

Breillout F, Antoine E and Poupon MF (1990) Methionine dependency of malignant tumours: a possible approach for therapy. *J Natl Cancer Inst* **82**: 1628–1632

Colombani F, Cherest H and De-Robichon-Szulmajster H (1975) Biochemical and regulatory effects of methionine analogues in *Saccharomyces cerevisiae*. *J Bacteriol* **122**: 375–384

Cox R and Irving CC (1977) Inhibition of DNA methylation by *S*-adenosylethionine with the production of methyl-deficient DNA in regenerating rat liver. *Cancer Res* **37**: 222–225

Crawford ED, Eisenberger MA, McLeod DC, Spaulding J, Benson R, Dorr FA, Blumenstein BA, Davis MA and Goodman PJ (1989) A control randomized trial of leuroprolide with and without flutamide in prostatic cancer. *N Engl J Med* **321**: 419–424

Fiskerstrand T, Christensen B, Tysnes OB, Ueland PM and Refsum H (1994) Development and reversion of methionine dependence in a human glioma cell line: relation to homocysteine remethylation and cobalamin status. *Cancer Res* **54**: 4899–4906

Gaudard-De Weck D (1989) Biodisponibilité de l'homocyst(é)ine comme précurseur de la méthionine chez le rat et l'homme. Perspectives pour l'alimentation du cancéreux. PhD thesis, Faculté des Sciences, Lausanne, Switzerland

Guo HY, Herrera H, Groce A and Hoffman RM (1993*a*) Expression of the biochemical defect of methionine dependence in fresh patient tumours in primary histoculture. *Cancer Res* **53**: 2479–2483

Guo HY, Lishko VK, Herrera H, Groce A, Kubota T and Hoffman RM (1993*b*) Therapeutic tumour-specific cell cycle block induced by methionine starvation *in vivo*. *Cancer Res* **53**: 5676–5679

Guo HY, Kubota T and Hoffman RM (1994) Anti-methionine cancer therapy: ethionine is synergistic with methionine depletion for inducing a therapeutic tumour-selective pre-mitotic cell-cycle block. *Proc Am Assoc Cancer Res Annu Meeting* **35**: 469

Hoffman RM (1993) Unchecked DNA synthesis and blocked cell division induced by methionine deprivation in a human prostate cancer cell line. *In Vitro Cell Dev Biol* **29A**: 359–361

© Cancer Research Campaign 1997 

Hoffman RM and Erbe R (1976) High *in vivo* dependency of methionine biosynthesis in transformed human and malignant rat cells auxotrophic for methionine. *Proc Natl Acad Sci USA* **73**: 1523–1527

Hoffman RM and Jacobsen SJ (1980) Reversible growth arrest in simian virus 40-transformed human fibroblasts. *Proc Natl Acad Sci USA* **77**: 7306–7310

Judde JG, Ellis M and Frost P (1989) Biochemical analysis of the role of transmethylation in the methionine dependence of tumour cells. *Cancer Res* **49**: 4859–4865

Kaignh ME, Narayan DS, Ohnuki Y, Lechner JF and Jones IW (1979) Establishment and characterization of a human prostatic carcinoma cell line (PC-3). *Invest Urol* **17**: 16–23

Kim HO, Balcezak TJ, Nathin SJ, McMullen HF and Hansen DE (1992) The use of a spectrophotometric assay to study the interaction of *S*-adenosylmethionine synthetase with methionine analogues. *Anal Biochem* **207**: 68–72

Kramer DL, Porter CW, Borchardt RT and Sufrin JR (1990) Combined modulation of *S*-adenosylmethionine biosynthesis and *S*-adenosylhomocysteine metabolism enhances inhibition of nucleic acid methylation and L1210 cell growth. *Cancer Res* **50**: 3848–3842

Lasserre C, Sabatier L, Beaumatin J, Luccioni C, Lefrançois D, Muleris M and Dutrillaux B (1994) Gene dosage and expression, and enzyme activity of thymidine kinase and thymidilate synthase in xenografted colorectal adenocarcinomas. *Int J Cancer* **56**: 506–511

Levine M and Tarver H (1951) Studies on ethionine. III. Incorporation of ethionine into rat proteins. *J Biol Chem* **187**: 835–850

Liteplo RG, Hipwell SE, Rosenblatt DS, Sillaots S and Lue-Shing H (1991) Changes in cobalamin metabolism are associated with the altered methionine auxotrophy of highly growth autonomous human melanoma cells. *J Cell Physiol* **149**: 332–338

Mecham JO, Rowitch D, Wallace CD, Stern PH and Hoffman RM (1983) The metabolic defect of methionine dependence occurs frequently in human tumour cell lines. *Biochem Biophys Res Commun* **117**: 429–434

Miccoli L, Oudard S, Sureau F, Poirson F, Dutrillaux B and Poupon MF (1996) Intracellular pH governs the subcellular distribution of hexokinase in a glioma cell line. *Biochem J* **313**: 957–962

Ogier G, Chantepie J, Deshayes C, Chantegrel B, Charlot C, Doutheau A and Quash G (1993) Contribution of 4-methylthio-2-oxobutanoate and its transaminase to the growth of methionine-dependent cells in culture. *Biochem Pharmacol* **45**: 1631–1644

Oudard S, Poirson F, Miccoli L, Bourgeois Y, Vassault A, Poisson M, Magdelenat H, Dutrillaux B and Poupon MF (1995) Mitochondria-bound hexokinase as target for therapy of malignant gliomas. *Int J Cancer* **62**: 216–222

Pezacka EH, Jacobsen DW, Luce K and Green R (1992) Glial cells as a model for the role of cobalamin in the nervous system: impaired synthesis of cobalamin coenzymes in cultured human astrocytes following short-term cobalamin deprivation. *Biochem Biophys Res Commun* **184**: 832–839

Poirson F, Lopez R, Monneret C, Dutrillaux B and Poupon MF (1996) Methionine analogs inhibit cell proliferation and growth of human xenografted gliomas. *Proc Am Assoc Cancer Res* **37**: 399

Poupon MF, Arvelo F, Goguel AF, Bourgeois Y, Jacrot M, Hanania N, Arriagada R and Chevalier TL (1993) Response of small-cell lung cancer xenografts to chemotherapy: multidrug resistance and direct clinical correlates. *J Natl Cancer Inst* **85**: 2023–2029

Redgate ES, Boggs S, Grudziak A and Deutsch M (1995) Polyamines in brain tumour therapy. *J Neurooncol* **25**: 167–179

Simons JW (1994) Genetically modified tumour vaccines for prostate cancer therapy. *Proc Am Assoc Cancer Res Annu Meeting* **35**: 676

Stern PH and Hoffman RM (1986) Enhanced in vitro selective toxicity of chemotherapeutic agents for human cancer cells based on a metabolic defect. *J Natl Cancer Inst* **76**: 629–639

Warburg O (1956) On the origin of cancer cells. *Science* **123**: 309–314

Wingo PA, Tong T and Bolden S (1995) Cancer statistics. *CA Cancer J Clin* **45**: 8–30

Wyllie AH (1992) Apoptosis and the regulation of cell numbers in normal and neoplastic tissues: an overview. *Cancer Metas Rev* **11**: 95–103

 *© Cancer Research Campaign 1997*